# Recent Patents of Interest to Dentists

**Published:** 1907-11

**Authors:** 


### PATENTS.

RECENT PATENTS OF INTEREST TO DENTISTS.

867017. Tooth Powder Receptacle, Herman L. DeMond, Philadelphia.
867193. Dental Soldering Apparatus, Edward Flannigain, St. Louis.
867062. Tooth Brush, Orville C. Lille, Conneaut, Ohio.
867063. Rotary Tooth Brush, Orville C. Lillie, Conneaut, Ohio.
866962. Swage for Dental Crown Plates and Similar Articles, Carl Rauhe, Dusseldorf, Germany.
866963. Dental Bur, Carl Rauhe, Dusseldorf, Germany.
866753. Head Rest, Henry E. Weber, Canton, Ohio.
867264. Dental Implement, Charles S. Evans, Dayton, Ohio.
868628. Dental Regulator and Spacer, Eben M. Flagg, New York, N. Y.
868109. Making Artificial Teeth, Joseph Morris, North Wales, Pa.

Copies of above patents may be obtained for fifteen cents each, by addressing John A. Saul, Solicitor of Patents, Fendall Building, Washington, D. C.

